# IL-17+ CD8+ T cells: Differentiation, phenotype and role in inflammatory disease

**DOI:** 10.1016/j.imlet.2016.05.001

**Published:** 2016-10

**Authors:** Ushani Srenathan, Kathryn Steel, Leonie S. Taams

**Affiliations:** Centre for Molecular and Cellular Biology of Inflammation, Division of Immunology, Infection & Inflammatory Disease, King’s College London, London, UK

**Keywords:** EAE, experimental autoimmune encephalomyelitis, HCV, hepatitis C virus, IRF, interferon regulatory factor, RORγ, retinoic acid receptor-related orphan receptor gamma, CD8+, IL-17, Tc17, Inflammation, Disease

## Abstract

The pro-inflammatory cytokine interleukin-17A (IL-17) has been the subject of research by many groups worldwide. IL-17 expression is often associated with a specific subset of CD4+ T cells (the so-called Th17 cells); however various other immune cell subsets can also synthesise and express IL-17, including CD8+ T cells. Here we review recent data regarding the presence of IL-17+ CD8+ T cells (also known as Tc17 cells) in human inflammatory disease, discuss current knowledge regarding the culture conditions required for the differentiation of these cells in humans and mice, and describe key phenotypic and functional features. Collectively, this information may shed light on the potential pathogenic role that IL-17+ CD8+ T cells may play in human inflammatory disease.

## Highlights

• IL-17A (IL-17) is produced by multiple cell subsets, including CD8+ T cells.
• The presence of IL-17+ CD8+ T cells in human inflammatory diseases suggests these cells may contribute to immunopathology.
• Increased knowledge of human IL-17+ CD8+ T cells will enhance our overall understanding of their role in human disease.

## 1 Introduction

Since its discovery in 1993 [1], the pro-inflammatory cytokine interleukin (IL)-17A (in this review generally referred to as IL-17) has been the subject of intense research. The interest in this cytokine increased considerably when it was found to be produced by a specific subset of CD4+ T cells, the so-called Th17 cells. However, it is well established that other immune cell subsets can also synthesise and express IL-17, including CD8+ T cells. In this review, we summarise current data regarding the presence of IL-17+ CD8+ T cells in human inflammatory disease, discuss the differentiation and polarisation protocols reported to induce these cells in humans and mice, and describe current knowledge regarding their phenotype and function. We also discuss how these cells may contribute to immunopathology in human inflammatory diseases.

## 2 Presence of IL-17+ CD8+ T cells in human inflammatory disease

The presence of IL-17-expressing CD8+ T cells (also referred to as Tc17 cells) has been described in several human inflammatory diseases. An early study reported the presence of IL-17 mRNA in CD8+ T cell clones derived from psoriatic lesional skin [2]. Later studies using flow cytometry, demonstrated that psoriatic skin plaques contain increased numbers [3] or proportions of IL-17+ CD8+ T cells [4], [5], [6], whilst this was not observed in control skin samples. Our own lab showed that synovial fluid from the inflamed joints of patients with psoriatic arthritis, but not rheumatoid arthritis, contains increased frequencies of IL-17+ CD8+ T cells compared to matched peripheral blood [7]. In active lesions in brain tissue from patients with multiple sclerosis, IL-17 expression was detected in both CD8+ and CD4+ T cells with equal distribution, and both cell types were present at higher levels compared to inactive lesions [8]. In children with new onset type I diabetes, an increased percentage of IL-17+ cells within peripheral blood CD8+ and CD4+ T cell populations was found following 3 days of *in vitro* stimulation compared to age-matched healthy controls [9]. IL-17+ CD8+ T cells were found to be enriched in the liver of patients with chronic hepatitis C virus (HCV) infection or nonalcoholic steatohepatitis [10] and in the pleural effusion of tuberculosis patients [11] compared to peripheral blood. Finally, using immunofluorescence staining, CD8+ T cells expressing IL-17A and IL-17F were detected in bronchoscopic biopsies from the subsegmental bronchi of patients with chronic obstructive pulmonary disease, at percentages similar to CD4+ T cells [12]. Together, these data demonstrate that IL-17+ CD8+ T cells are present in inflamed tissue in various human inflammatory diseases suggesting these cells may contribute to immune pathology.

## 3 IL-17+ CD8+ T cell differentiation and polarisation in humans and mice

It is well established that transforming growth factor (TGF)-β, IL-6, IL-1β, IL-21 and IL-23 can promote IL-17+ CD4+ T cell differentiation in humans [13], [14], [15], [16] and mice [17], [18], [19], [20]. Since IL-17+ CD8+ T cells have a similar cytokine profile to IL-17+ CD4+ T cells, this provides a rationale for applying IL-17+ CD4+ T cell polarising conditions to induce or expand IL-17+ CD8+ T cells. Table 1 summarises the *in vitro* culture conditions reported thus far to expand human or mouse IL-17+ CD8+ T cells and IL-17+ interferon (IFN)-γ+ dual producing CD8+ T cells. A limited number of human IL-17+ CD8+ T cell differentiation studies are published to date compared to those in mice. One study reported that human IL-17+ CD8+ T cells were induced upon culture of naïve CD8+ T cells with recombinant TGF-β, IL-6, IL-1β, IL-23 and α-IFN-γ mAb for 5 days, followed by IL-2 addition for a further 4 days [21]. However, a representative figure showed 0.11% of IL-17+ CD8+ T cells indicating that only a limited percentage of these cells was induced. Another protocol involved culturing human bulk CD8+ T cells with TGF-β and IL-6 for 3 days [22]. IL-17+ CD8+ T cell induction frequencies were not reported, but low IL-17 levels were detected by ELISA.

More detailed information stems from mouse studies, in which TGF-β and IL-6 have been used to drive IL-17+ CD8+ T cell differentiation from CD8+ T cells [23], [24], [25], [26], [27], [28], [29], leading to frequencies ranging from 19%–64% (Table 1). TGF-β decreases IFN-γ production, while reducing cytolytic activity and expression of the cytolytic marker granzyme B within *in vitro* cultured CD8+ T cells [24], [25]. TGF-β also inhibits CD8+ T cell proliferation and division, but in concert with IL-6, these TGF-β-mediated actions are opposed while maintaining reduced cytolytic activity, a characteristic of IL-17+ CD8+ T cells [25]. A role for IL-6 in IL-17+ CD8+ T cell induction was also shown in mice *in vivo*, since after allogeneic stem cell transplantation IL-6R blockade reduced IL-17+ CD8+ T cell frequencies [30]. In contrast to IL-6, the effects of TGF-β may vary between *in vitro* and *in vivo* conditions. TGF-β removal from the IL-17+ CD8+ T cell differentiation cocktail containing IL-1β, IL-2, IL-6, IL-21, IL-23, α-IL-4 and α-IFN-γ mAbs led to a strong reduction in IL-17+ CD8+ T cell percentages *in vitro* [23]. However, *in vivo* TGF-β neutralisation in mice did not considerably affect IL-17+ CD8+ T cell frequencies [30]. Furthermore, TGF-βRIIDN mice with impaired TGF-β signalling still exhibited IL-17+ CD8+ T cell differentiation, whilst IL-17+ CD4+ T cell differentiation was inhibited [31], suggesting that TGF-β may not be critical for *in vivo* IL-17+ CD8+ T cell differentiation, and that cytokines required for IL-17 induction in CD4+ versus CD8+ T cells may differ.

IL-21 has also been shown to be important for IL-17+ CD8+ T cell differentiation in mouse cells, either as part of a cytokine cocktail (TGF-β, IL-6, IL-1β, IL-2, IL-21, IL-23, α-IL-4 and α-IFN-γ mAb) [23] or in combination with TGF-β [32]. Increased *Il21* mRNA expression was observed in mouse CD8+ T cells cultured with TGF-β and IL-21, with TGF-β and IL-6, or with IL-21 alone [24]. IL-21 production by IL-17+ CD8+ T cells may promote a positive feedback loop to expand IL-17+ CD8+ T cells further, an autocrine mechanism reported in IL-17+ CD4+ T cells [20], [33]. Human stimulated IL-17+ CD8+ T cells from psoriatic lesions express IL-21 [5], however it remains to be established whether IL-21 is important for human IL-17+ CD8+ T cell differentiation.

IL-23 is often used to expand human IL-17+ CD4+ T cells [14], [15]. IL-23 addition to hapten-primed mouse CD8+ T cell and dendritic cell co-cultures induced IL-17 production [34], yet IL-23 alone only slightly induced *Il17a* expression in mouse naïve CD8+ T cell cultures [27]. Thus, IL-23 may maintain the IL-17+ CD8+ T cell phenotype rather than drive differentiation, similar to its role in IL-17+ CD4+ T cells [35]. In humans, a role for IL-23 in IL-17+ CD8+ T cell differentiation is not yet elucidated, however one study revealed that heterozygous carriers of the R381Q IL23R variant exhibited reduced IL-17+ CD8+ T cell frequencies compared to carriers of the common variant [36], indicating a potential role of IL-23 in human IL-17+ CD8+ T cell development. IFN-γ neutralisation expanded mouse IL-17+ CD8+ T cells *in vitro* [23], [26], [32] and α-IFN-γ mAb removal from the polarising cocktail also containing IL-1β, IL-2, IL-6, IL-21, IL-23, TGF-β and α-IL-4 mAb, reduced IL-17+ CD8+ T cell frequencies [23]. These data indicate that IFN-γ reduces IL-17+ CD8+ T cell expansion, as seen in mouse IL-17+ CD4+ T cell studies [37]. In support of this, Type I IFN signalling-deficient mice (used to inhibit IFN-γ+ CD8+ T cell induction) treated with neutralising IFN-γ Abs showed higher IL-17+ CD8+ T cell levels *in vivo,* as compared to wild type mice [27]. IL-17+ CD8+ T cell frequencies were also expanded *in vivo* when allogeneic mice were injected with α-IFN-γ mAb 7 days post-stem cell transplant [30]. Additionally, higher IL-17+ CD8+ T cell frequencies were reported in IFN-γ-deficient OT-I mice compared to wild-type mice [23], further indicating that IFN-γ inhibition *in vivo* enhances IL-17 production by CD8+ T cells in mice. IFN-γ was neutralised in one *in vitro* human IL-17+ CD8+ T cell differentiation study [21], however further investigations are required to establish its exact role in the human context.

Collectively, the findings reported thus far indicate that similarities exist between the culture conditions used for mouse IL-17+ CD8+ and IL-17+ CD4+ T cell differentiation. However, there are still significant gaps in our knowledge regarding the exact conditions required for human IL-17+ CD8+ T cell induction or expansion. It will be important to address these gaps in future, given the accumulating evidence of the presence of these cells in human inflammatory disease, which warrants detailed investigation of their function.

## 4 Phenotype of IL-17+ CD8+ T cells in humans and mice

**Fig. 1:**
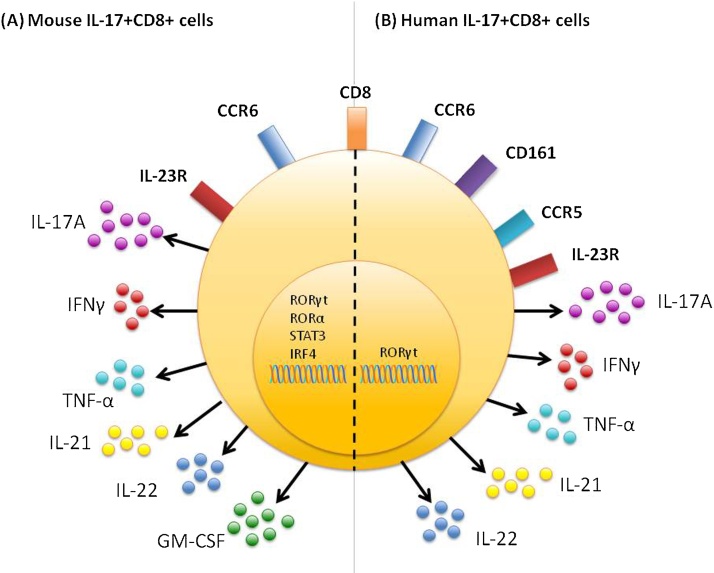
Phenotype of mouse and human IL-17+ CD8+ T cells. (A) Mouse IL-17+ CD8+ T cells express CCR6 and IL-23R [24], [26], and can produce the pro-inflammatory cytokines IL-17A, IFN-γ, TNF-α, IL-21, IL-22 and GM-CSF [24], [26], [29], [30]. Transcription factors expressed by mouse IL-17+ CD8+ T cells include RORγt, RORα, STAT3 and IRF4 [24], [26], [29]. (B) Human IL-17+ CD8+ T cells express CCR6, CD161, CCR5 and IL-23R [5], [7], [10], [21] and can produce IL-17A, IFN-γ, TNF-α, IL-21 and IL-22 [5], [21]. Expression of the transcription factor RORγt has been confirmed in human IL-17+CD8+ cells [5].

To date, phenotypic profiling of human IL-17+ CD8+ T cells has been limited at both protein and molecular level, although some characterisation has been performed. Furthermore, variation in the inflammatory sites from which cells are sourced combined with disparity between *in vitro* induction or expansion protocols makes comparison of individual studies challenging. Despite these challenges some phenotypic features have been described for human IL-17+ CD8+ T cells including surface marker, cytokine and transcription factor expression. A summary of current mouse and human IL-17+ CD8+ T cell phenotype data is shown in Fig. 1. Several of these features are shared with IL-17+ CD4+ T (Th17) cells, indicating some similarities between these cell types which may give an insight into the functional potential of IL-17+ CD8+ T cells

The most definitive feature of human IL-17+ CD8+ T cells is their ability to produce the pro-inflammatory cytokine IL-17A (IL-17) but concurrent expression of several other cytokines has been shown. The most well-described of these is the pro-inflammatory cytokine IFN-γ, which is found to be co-expressed with IL-17 in cultured IL-17+ CD8+ T cells from healthy blood [21] and psoriatic plaques [5]. Furthermore IL-17/IFN-γ dual producers derived from the liver tissue of patients with hepatitis C infection produced higher levels of IFN-γ than IFN-γ+ CD8+ T (Tc1) cells, indicating these cells may represent a population with higher pro-inflammatory potential [10]. In addition, several other pro-inflammatory cytokines such as tumour necrosis factor alpha (TNF-α), IL-21 and IL-22 have been shown to be co-expressed with IL-17 by human and mouse IL-17+ CD8+ T cells [5], [10], [24], [26]. Granulocyte macrophage colony stimulating factor (GM-CSF) co-expression has also been reported, at least in mouse [30]. Moreover, evidence for lack of co-expression of the anti-inflammatory cytokine IL-10 by IL-17+ CD8+ T cells in mice supports classification of IL-17+ CD8+ T cells as pro-inflammatory [30]. At the molecular level, studies into the phenotype of human IL-17+ CD8+ T cells have been restricted, with only one study showing that *RORC* (encoding retinoic acid receptor-related orphan receptor gamma; RORγt) gene expression was increased in psoriatic skin IL-17+ CD8+ T cells, compared to IL-17- CD8+ T cells, which in contrast expressed higher expression of *TBX21* (encoding T-Bet) [5]. This is supported by data from mouse models where *Rorc* has been shown to be expressed at a high level in spleen and lymph node (LN) derived IL-17+ CD8+ T cells differentiated *in vitro* [24], [26], [29]. RORγt has been extensively implicated in the development and function of IL-17+ CD4+ T cells, and these data suggest that IL-17+ CD8+ T cell phenotype and function may be defined by a similar mechanism.

In the above studies, *Rorc* up-regulation in IL-17+ CD8+ T cells was accompanied by an increase in expression of another ROR subfamily homologue, *Rora* (encoding retinoic acid receptor-related orphan receptor alpha) plus a reduction in *Eomes* (encoding Eomesodermin) [24], [26], [29], [30]. Other markers of CD8+ T cell effector cells such as *Tbx21* and *Gata3* were reduced in these cells with the exception of one study in which CD8+YFP+ cells from IL-17A-YFP+ reporter mice were shown to have notable *Tbx21* expression, although this was performed over an extensive time course and may be linked to the plasticity of IL-17+ CD8+ T cells to produce IFN-γ over time [30]. Furthermore, CD8+ T cells from *Tbx21* and *Eomes* double knockout mice have higher IL-17A expression compared to wild type cells [38]. Overall these findings indicate that the absence of *Tbx21* and *Eomes* expression plus up-regulation of *Rora* and *Rorc* currently represent a reliable molecular characterisation of IL-17+ CD8+ T cells, at least in mice.

Additional transcription factors have been implicated in the induction of IL-17+ CD8+ T cells including signal transducer and activator of transcription 3 (STAT3), interferon regulator factor 3 (IRF3) and interferon regulatory factor 4 (IRF4), at least in mice. Knockdown of STAT3 by siRNA in spleen or lymph node-derived CD8+ T cells resulted in a reduction of IL-17+ CD8+ T cell frequencies under IL-17+ CD8+ T cell differentiation conditions [24]. A similar reduction in IL-17+ CD8+ T cells was seen under these conditions in an IRF4 knockout mouse, and this phenotype was partially reversed upon overexpression of RORγt in IRF4−/− cells [29]. In contrast, CD8+ T cells derived from the spleen or lymph nodes of IRF3 knockout mice showed increased IL-17A production and upregulated *Il23r* gene expression under IL-17+ CD8+ T cell differentiation conditions. Interestingly, a direct interaction between IRF3 and RORγt transcription factors was identified in the cytoplasm under IL-17+ CD8+ T cell differentiation conditions indicating that IRF3 may act as a negative regulator of IL-17 induction in IL-17+ CD8+ T cells by antagonistically binding RORyt and therefore reducing downstream interactions [39].

At the protein level, human IL-17+ CD8+ T cells express several surface markers, although as yet no markers have been defined that are exclusively expressed on these cells. The majority of cultured IL-17+ CD8+ T cells isolated from active psoriatic plaques were found to express CD161 [5]. Furthermore, CD8+ T cells isolated from liver biopsies of patients with chronic HCV infection were found to co-express CD161 and IL-17 after *ex vivo* stimulation [10], which was also observed *ex vivo* in stimulated CD3+ CD4- IL-17+ T cells from the joints of patients with psoriatic arthritis [7]. CD161 has been shown to be a marker of CD4+ IL-17+ T lymphocytes, however it does not represent a definitive marker for IL-17+ T cells as not all CD161+ cells produce IL-17 [40].

IL-17+ CD8+ T cells have also been shown to express certain cytokine and chemokine receptors, highlighting their potential to respond to inflammatory mediators in the surrounding environment. When CD8+ T cells from healthy blood were sorted based on expression of CD8 and various chemokine receptors followed by PMA and ionomycin stimulation for 6 h, CCR5^high^ and CCR6+ populations were found to contain increased frequencies of IL-17+ CD8+ T cells compared to CCR4+ and CCR7+ cells [21]. Additionally, stimulated CD8+ T cells from psoriatic plaques, pleural effusions from patients with tuberculosis and healthy blood have been shown to co-express both IL-17 and CCR6 [11], [41]. Although these data do not confirm that all IL-17+ CD8+ T cells express CCR6, data from an IL-17A-YFP reporter mouse have shown an increase in *Ccr6* gene expression in YFP+CD8+ T cells indicating the expression of both CCR6 and IL-17A production is correlated to some extent [30]. With regards to cytokine receptor expression, IL-23 receptor (IL-23R) has been shown to be expressed on CCR6+ CD8+ T cells cultured under IL-17+ CD8+ T cell induction conditions [5]. This is further supported by an increase in IL-23R expression in IL-17+ CD8+ T cells derived from experimental autoimmune encephalomyelitis (EAE) mice [24].

Not unexpectedly, human IL-17+ CD8+ T cells appear to have a memory phenotype since the highest proportion of IL-17+ CD8+ T cells found in healthy blood are either CD27+CD28+CD45RA- or CD27-CD28+CD45RA- cells, and cultured IL-17+ CD8+ T cells from psoriatic skin express CD45RO [5], [11], [21].

## 5 Function and pathogenicity of IL-17+ CD8+ T cells

### 5.1 Production of cytokines

IL-17+ CD8+ T cells are defined by their ability to produce the pro-inflammatory cytokine IL-17A. These cells often also co-express one or several other cytokines including IFN-γ, TNF-α, IL-21, IL-22 and/or GM-CSF (the latter has not yet been reported in humans) [5], [10], [21], [26], [30]. IL-17 can induce production of inflammatory mediators such as TNF-α, IL-6, IL-8 and chemokine (C-X-C motif) ligand 1 (CXCL1) by monocytes and fibroblasts, induce production of matrix metalloproteinases and promote osteoclastogenesis [42]. IFN-γ, most frequently co-expressed in IL-17+ CD8+ T cells [5], [10], [21], is a pro-inflammatory cytokine typically expressed by some CD4+ and CD8+ T cells as well as some Natural Killer (NK) cells. IFN-γ is crucial for the induction of type I immunity and as described previously, IL-17A+ IFN-γ+ CD8+ T cells were found to produce higher levels of IFN-γ than IFN-γ single-producing T cells [10]. TNF-α is also expressed by a broad range of immune cell types during inflammation and is capable of acting on a number of cell types including synergistically with IL-17 to promote activation of synovial fibroblasts and keratinocytes in the skin and joint of patients with RA and psoriasis [43], [44]. IL-21 is a known driver of IL-17+ CD4+ T cell differentiation and is required for IL-17+ CD4+ T cell mediated pathogenesis in some models of inflammation in mice [33]; this cytokine may have a similar role in IL-17+ CD8+ T cell differentiation, though this is still to be fully investigated. IL-22 is produced by T cells and NK cells and has been shown to mediate keratinocyte differentiation and proliferation in psoriatic skin [45], [46]. GM-CSF is a potent driver of inflammation via its actions on myeloid cells at the site of inflammation [47]. Overall, the potential production of a broad range of pro-inflammatory cytokines by IL-17+ CD8+ T cells indicates these cells may contribute to pathogenesis via activation of neighbouring haematopoietic and stromal cells.

### 5.2 Plasticity

Cytokine heterogeneity and plasticity are characteristic features of CD4+ IL-17+ T cells [45], [48], and IL-17+ CD8+ T cell plasticity has been investigated in mouse studies. Adoptive transfer of *in vitro* generated and sorted IL-17+ CD8+ T cells, IFN-γ+ CD8+ T cells and IL-17+ IFN-γ+ CD8+ T cells to C3HA^high^ mice, which express haemagglutinin as a self antigen, resulted in a persistence of IL-17+ CD8+ T cells and IL-17+ IFN-γ+CD8+T cells but a loss of IFN-γ+ CD8+ T cells in recipient lungs after 7 days [26]. Adoptive transfer of IL-17-polarised premelanosome protein-1 specific CD8+ T cells to mice with melanoma led to a conversion of the IL-17+ CD8+ T cells towards an IFN-γ+ CD8+ T cell phenotype from days 5–11, with a small presence of dual-producing CD8+ T cells [49]. The most significant IFN-γ expression was observed within transferred IL-17+ CD8+ T cells, indicating plasticity of these cells *in vivo*. Culture of naive CD8+ T cells from IL-17A-EGFP reporter mice with IL-12 resulted in an IFN-γ+ cell subset within EGFP+ cells, confirming that IL-17+ IFN-γ+ CD8+ T cells can derive from IL-17+ CD8+ T cells *in vivo* [50]. A recent study utilising reporter IL-17A-YFP mice investigated the *in vivo* cytokine profile of CD8+ YFP+ T cells 7 and 21 days post-allogeneic stem cell transplant [30]. Cytokine profile heterogeneity with expression of IL-13, IL-22, TNF-α, GM-CSF and IFN-γ and minimal IL-10 expression was observed at day 7, and at 21 days CD8+ YFP+ cells possessed a pro-inflammatory cytokine profile, predominantly co-expressing IFN-γ, TNF-α and GM-CSF.

### 5.3 Cytotoxicity

IL-17+ CD8+ T cells are mostly characterised as non-cytotoxic in mice [23], [24], [25], [26], [29] and humans [7], [10] thus distinguishing these cells from IFN-γ+ CD8+ T cells. However, there are data implicating the cytotoxic function of IL-17+ CD8+ T cells in both mice [32] and humans [5]. Additionally, IL-12-converted mouse IL-17+ IFN-γ+ CD8+ T cells possessed more cytotoxic activity than IL-17-expressing CD8+ T cells [50], [51] and like IFN-γ+ CD8+ T cells, possessed anti-tumour functions [50]. Thus, variability in cytotoxicity may indicate functional differences of IL-17-expressing CD8+ T cells; however it remains to be determined whether and how (lack of) cytotoxic activity contributes to human inflammatory disease.

### 5.4 Pathogenicity

The pathogenicity of IL-17+ CD8+ T cells has been mainly investigated using transgenic mouse models. Using the OT-I transgenic model, antigen-specific IL-17+ CD8+ T cells treated with IL-23 were found to be diabetogenic when adoptively transferred into RIP-mOVA mice. The pathogenicity was diminished upon treatment with anti-IL-17A and anti-IL-17F antibodies, indicating that IL-17 is essential for disease development [32]. Furthermore, in EAE mice myelin oligodendrocyte glycoprotein-specific CD8+ T cells isolated from the lymph nodes and central nervous system at the peak of disease were found to express IL-17 *ex vivo* (after PMA and ionomycin stimulation). These cells did not express granzyme B, indicating their potential pathogenicity was not dependent on a cytotoxic mechanism and may be related to pro-inflammatory cytokine production [24]. As previously mentioned, healthy individuals carrying the protective R381Q variant in the IL23R locus had a lower IL-17+ CD8+ T cell frequency in peripheral blood compared to those who did not carry the variant [36]. Since carriage of this variant (or others in high linkage disequilibrium with R381Q) confers decreased susceptibility to immune mediated diseases such as inflammatory bowel disease, ankylosing spondylitis and psoriasis [52], [53], [54], this indicates a potential unidentified role of IL-17+ CD8+ T cells in the pathogenesis of these diseases. In the context of psoriatic arthritis, CD8+ IL-17+ T cells frequencies were increased in the synovial fluid compared to peripheral blood, correlated with several clinical parameters of disease and were associated with erosive disease, suggesting these cells may play a role in the pathogenesis of this disease [7].

## 6 Concluding remarks

A growing evidence base indicates the presence of IL-17+ CD8+ T cells at sites of inflammation in humans. These cells bear resemblance to their IL-17+ CD4+ T cell counterparts in terms of phenotypic markers and cytokine co-expression typically associated with Th17 cells. The exact requirements for differentiation and/or polarisation of IL-17+ CD8+ T cells are less well-defined, particularly in humans. Future in-depth phenotypic, molecular and functional characterisation of these cells will help determine how IL-17+ CD8+ T cells may contribute to human inflammatory disease.

## Figures and Tables

**Table 1:** Summary of reported culture conditions used to induce or expand human and mouse IL-17+ CD8+ T cells *in vitro*.

Species	CD8+ T cell type	Culture conditions	IL-17+ CD8+ T cell yield	IL-17+ IFN-γ+ CD8+ T cell yield	Ref.
Human	Naïve CD8+ T cells + α-CD3 + α-CD28	TGF-β, IL-6, IL-1β, IL-23 and α-IFN-γ (5 days), IL-2 (further 4 days).	0.11% of naïve CD8+ T cells expressed IL-17 (representative figure using intracellular staining)	N/A	[21]

	Bulk CD8+ T cells + α-CD3 + α-CD28	TGF-β and IL-6 (3 days)	No report on % IL-17+ CD8+ T cells. 25 pg/ml IL-17A secretion by ELISA (n = 3)	N/A	[22]

Mouse	Lymph node hapten-primed CD8+ T cells + bone-marrow derived dendritic cells	IL-23 (2 days)	No report on % IL-17+ CD8+ T cells. 1.4 ng/ml IL-17A secretion by ELISA (n = 3)	N/A	[34]

	Mixed lymphocyte cultures (splenocytes)	TGF-β and IL-6 (5 days)	32% of CD8+ cells expressed IL-17 (representative figure using intracellular staining, reported to be reproducible in n = 3)	N/A	[25]

	Naïve CD8+ T cells (spleen or lymph node) + Ag + α-CD3 + α-CD28	TGF-β and IL-6 or TGF-β and IL-21 (3 days)	TGF-β + IL-6: 29% of CD8+ T cells expressed IL-17	TGF-β + IL-6: 1.2% CD8+ T cells expressed IL-17 and IFN-γ	[24]
			TGF-β + IL-21: 24% of CD8+ T cells expressed IL-17 (representative figure using intracellular staining)	TGF-β + IL-21: 0.7% CD8+ T cells expressed IL-17 and IFN-γ (representative figure using intracellular staining)	

	OT-I CD8+ T cells stimulated with OVA-derived peptide SIINFEKL-pulsed B blasts	TGF-β, IL-6, IL-1β, IL-2, IL-21, IL-23, α-IL-4 and α-IFN-γ (4 days)	54% of CD8+ T cells expressed IL-17 (representative figure using intracellular staining)	0.5% of CD8+ T cells expressed IL-17 and IFN-γ (representative figure using intracellular staining)	[23]

	Naïve CD8+ T cells (splenocytes) activated with cognate peptide and irradiated antigen-presenting cells (APCs)	TGF-β, IL-6, IL-1β, IL-23, α-IL-4 and α-IFN-γ (2 days), followed by a 3 day rest.	39% (mean) of CD8+ T cells expressed IL-17 (n = 4)	4% (mean) of CD8+ T cells expressed IL-17 and IFN-γ (n = 4)	[26]

	Bulk CD8+ T cells (splenocytes) stimulated with OVA-peptide + IL-12/IL-23p40 deficient APCs	TGF-β and IL-6 (5 days)	23% (mean) of CD8+ T cells expressed IL-17 (n = 4)	N/A	[27]

	Bulk CD8+ T cells (splenocytes) + α-CD3 + α-CD28	Combinations of cytokines: TGF-β, IL-6, IL-21, IL-23, IL-1β, TNF-α, IL-2, α-IFN-γ, α-IL-2 (3 days)	TGF-β + IL-6: 45% of CD8+ T cells expressed IL-17 TGF-β, IL-6, α-IFN-γ, α-IL-2: 64% of IL-17-expressed CD8+ T cells (based on representative figures using intracellular staining)	TGF-β + IL-6: 10% of CD8+ T cells expressed IL-17 and IFN-γ TGF-β, IL-6, IL-1β: 18% of CD8+ T cells expressed IL-17 and IFN-γ (based on representative figures using intracellular staining)	[32]

	Bulk CD8+ T cells + α-CD3 + α-CD28 and CD8+-depleted irradiated splenocytes	TGF-β and IL-6 (5 days)	57% of CD8+ T cells expressed IL-17 (based on representative figures using intracellular staining)	16% of CD8+ T cells expressed IL-17 and IFN-γ (based on representative figures using intracellular staining)	[28]

	Bulk CD8+ T cells + α-CD3 + α-CD28	TGF-β and IL-6 (3 days)	TGF-β + IL-6: 19% of CD8+ T cells expressed IL-17 (based on representative figures using intracellular staining)	N/A	[29]

Table 1 summarises data from existing literature regarding *in vitro* induction protocols of mouse and human IL-17+ CD8+ T cells. The table lists the cell type, TCR stimulation and co-stimulation methods, recombinant cytokines and blocking mAbs used, culture duration and yield of both IL-17+ CD8+ T cells and IL-17+ IFN-γ+ dual producing CD8+ T cells.
